# Microchip gel electrophoretic analysis of perchloric acid‐soluble serum proteins in systemic inflammatory disorders

**DOI:** 10.1002/elps.201800378

**Published:** 2018-11-16

**Authors:** Lilla Makszin, Péter Kustán, Balázs Szirmay, Csilla Páger, Emerencia Mező, Krisztina I. Kalács, Vera Pászthy, Erzsébet Györgyi, Ferenc Kilár, Andrea Ludány, Tamás Kőszegi

**Affiliations:** ^1^ Institute of Bioanalysis Medical School University of Pécs Pécs Hungary; ^2^ Department of Laboratory Medicine Medical School University of Pécs Pécs Hungary; ^3^ János Szentágothai Research Centre University of Pécs Pécs Hungary

**Keywords:** Acid‐soluble serum proteins, Inflammation, Microchip gel electrophoresis, Perchloric acid precipitation, Systemic inflammatory diseases

## Abstract

Perchloric acid (PCA) precipitation is a well‐known method for the separation of heavily glycosylated proteins and for reducing the masking effect of major serum proteins. The aim of this study is to characterize PCA‐soluble serum proteins in healthy individuals and in patients with systemic inflammatory diseases, such as Crohn's disease and sepsis. A PCA precipitation protocol was prepared and adapted to the analytical methods. After PCA treatment of the serum, the soluble proteins in the supernatant were analyzed by SDS‐PAGE and by microchip gel electrophoresis (MGE). Characteristic changes of the electrophoretic patterns of the PCA‐soluble fractions were observed. Four characteristic bands (at ∼11, ∼65, ∼85, and ∼120 kDa) with varying intensity were detected by MGE. The proportion of the ∼65, ∼85, and ∼120 kDa bands were significantly higher in systemic inflammatory conditions than in healthy individuals (*p* < 0.001), and characteristic patterns were observed in patients with acute inflammation. The marked differences in the acid‐soluble protein patterns, which were observed in patients with ongoing systemic inflammation, might be a good indicator of inflammation. The MGE analysis is a fast screening and quantification method for the detection of characteristic changes among acid‐soluble serum proteins.

**Abbreviations**
CD: Crohn's disease
hs‐CRP: high sensitivity C‐reactive protein
MGE: microchip gel electrophoresis
ORM: orosomucoid
PCA: perchloric acid
WBC: white blood cell

## 1 Introduction

Laboratory analyses are essential for the diagnosis and monitoring of systemic inflammatory diseases; however, conventional laboratory tests have limited specificity and sensitivity, therefore, the identification of disease‐specific molecules is of utmost importance 1, 2. The roles of several proteins present in low amounts in the serum are still unrevealed because the masking effect of major serum proteins generates difficulties in the analysis 3.

Many assays require removal of the abundant proteins from the samples prior to analysis. Perchloric acid (PCA) precipitation is one of the most extensively used deproteinization protocols; however, certain proteins and small molecules might remain soluble 4. PCA is used for the denaturation and precipitation of major serum proteins except for glycoproteins and for alkaline proteins, which remain soluble. PCA precipitation is a traditional method to solubilize and isolate serum mucoproteins—heavily glycosylated serum proteins—to enrich glycoproteins in serum samples and to remove redundant proteins from the samples 5, 6. This method provides a useful tool to prepare samples for the analysis of a variety of small molecules and glycoproteins as potential biomarkers of disease progression 1, 4, 5.

Acid‐soluble serum proteins have been studied, mostly in malignant diseases 7. Among others, the spectra of proteins in breast cancer 8 and lung cancer 9, 10 were studied by PCA extraction. In early stage breast cancer, mucin‐type O‐glycosylated proteins were identified 8. Experiments were conducted to detect acid‐soluble proteins not only in human tumor diagnostics but also in rats with an underactive or overactive thyroid gland. In animals with hyperthyroidism, the amount of proteins soluble in PCA was elevated, and the level of protein‐bound hexoses was also higher. In the case of hypothyroidism, the total glycoprotein content decreased 11.

Low molecular mass human serum proteins, peptides, and other small components have been associated with pathological conditions such as cancer 12, diabetes 13, and cardiovascular and infectious diseases 14, most likely reflecting the state of the underlying cells or tissues.

Several inflammatory markers and acute phase proteins (orosomucoid, haptoglobin, alpha‐1‐anti trypsin, etc.) as glycoproteins might occur in the PCA‐soluble serum fraction 2, 4, 5. These glycoproteins could provide clinically relevant information for the early recognition and monitoring of systemic inflammatory conditions, which may improve the outcome and may predict progression.

Glycoproteomics has been chosen as a tool for the investigation of novel diagnostic possibilities 15, 16; however, besides the mass spectrometry‐based methods, different electrophoretic techniques are commonly used in daily routine diagnostics. Specific electrophoretic patterns might also serve as useful parameters for the assessment of disease activity 17, 18.

Beside SDS‐PAGE, microchip gel electrophoresis (MGE) was the method to separate and characterize glycoprotein samples according to their molecular mass. Engel et al. analyzed five proteins using the MGE method: bovine alpha‐1‐acid glycoprotein, human alpha‐1‐antitrypsin, recombinant human erythropoietin beta, hen egg white ovalbumin, and human serum transferrin with different degrees and patterns of glycosylation, and the results were compared to SDS‐PAGE. Their applied MGE method is highly standardized, rapid, and sensitive for glycoprotein analysis 19.

The aim of this study is to optimize the PCA precipitation method of serum proteins for MGE measurements, to investigate the electrophoretic profiles of PCA‐soluble serum fraction in systemic inflammatory conditions, and to identify inflammation‐specific patterns, which may be applicable in the detection of different inflammatory states.

## 2 Materials and methods

### 2.1 Sample collection

Several patient groups with acute and chronic inflammatory disorders were studied to reveal characteristic patterns in the PCA‐soluble protein fraction of the serum. Septic patients admitted to the intensive care unit (Department of Anesthesiology and Intensive Therapy, University of Pécs) served as severe acute inflammatory group (*n* = 56). Since Crohn's disease (CD) is considered to be a chronic inflammatory condition with altering activity, CD patients (*n* = 62) were enrolled (2^nd^ Department of Internal Medicine and Nephrology Centre, University of Pécs) as well. Based on clinical classification of CD patients, active (*n* = 28) and nonactive (*n* = 34) groups were created. Healthy volunteers served as controls (*n* = 25). Venous blood was obtained from the individuals using clot activator containing plain Vacutainer tubes (BD, Franklin Lakes, NJ, USA). After centrifugation of the coagulated blood (1500 × *g*, 10 min) the serum fractions were stored at –70°C until analyses.

The study protocol was approved by the Regional Ethics Committee of the University of Pécs, Hungary in accordance with the 2008 Helsinki declaration (Permission No.: 4327/KK/15/2011 and 5133/KK/15/2013). Written consent was obtained from all participants prior to sample collection. The patients’ parameters and demographic data are shown in Table 1. White blood cell (WBC) counts were determined using a Sysmex XN‐ 3000 automated hematology analyzer (Sysmex, Kobe, Japan); high sensitivity C‐reactive protein (hs‐CRP) and orosomucoid (ORM) concentrations were determined with a fully automated particle‐enhanced immune turbidimetric assay by Cobas 8000/c502 analyzer (Roche Diagnostics GmbH, Mannheim, Germany).

**Table 1:** Patients’ parameters (demographic data and routine serum tests)

	Controls (*n* = 25)	Nonactive CD patients (*n* = 34)	Active CD patients (*n* = 28)	Septic patients (*n* = 56)	*p*‐value
Males, *n* (%)	9 (36.0)	18 (52.9)	9 (32.1)	21 (37.5)	–
Age (years)	29 ± 16	26 ± 17	26 ± 16	67 ± 10 a, b, c	<0.001
Serum TP (g/L)	71 (68–74)	73 (69–77)	69 (65–73)	47 (42–51)^a^, ^b^, ^c^	<0.001
WBC count (G/L)	6.4 (4.9–7.7)	6.9 (6.0–8.9)	8.9 (6.5–12.3)^a^	12.7 (8.9–17.8)^a^, ^b^	<0.001
hs‐CRP (mg/L)	0.5 (0.2–1.8)	2.7 (0.9–6.1)	21.9 (7.1–71.5)^a^, ^b^	171.4 (91.9–247.4)^a^, ^b^, ^c^	<0.001
Orosomucoid (g/L)	0.8 (0.7–0.8)	1.0 (0.8–1.3)	1.7 (1.2–2.6)^a^, ^b^	1.8 (1.4–2.0)^a^, ^b^	<0.001

Medians (interquartiles) are presented except for age: mean ± SD.

TP, total protein; WBC, white blood cell; hs‐CRP, high sensitivity C‐reactive protein; CD, Crohn's disease; Kruskal‐Wallis test with post hoc analysis was used to compare groups. Superscript lowercase letters indicate the significance level of post hoc analysis.

a *p* < 0.01 compared to controls.

b *p* < 0.01 compared to nonactive CD.

c *p* < 0.01 compared to active CD.

### 2.2 Extraction of PA‐soluble proteins

The extraction of PCA‐soluble proteins was performed as it follows. Serum samples were mixed with an equal volume of 1.0 M PCA for 10 min at 4°C. Precipitated proteins were sedimented by centrifugation at 3800 × *g* for 6 min. Supernatant was collected and neutralized by adding 1.42 M NaOH in a ratio of 3:2. After 10 min incubation in room temperature, the mixture was centrifuged at 3800 × *g* for 6 min, and 2 M Tris‐HCl (pH 8.1) buffer was added to the supernatant in a ratio of 3.3:1. The final pH of the solution was between 8.5 and 8.8. The total protein concentration (serum total protein in Table 1 and total protein in Table 2) of the samples was determined by spectrophotometry at 220 nm (Hitachi U‐2910 UV/VIS). For UV absorbance measurements, samples were diluted ten times with distilled water. Bovine serum albumin standard solution was used for calibration (*y* = 0.0105*x* + 0.0076, *R* ^2^ = 1).

**Table 2:** MGE protein profiles of PCA‐soluble components (percent (%) of total AUC)

	Controls (*n* = 25)	Nonactive CD patients (*n* = 34)	Active CD patients (*n* = 28)	Septic patients (*n* = 56)	*p*‐value
TP (g/L)	1.2 (1.0–1.5)	1.7 (1.4–2.1)	2.9 (2.2–3.7)^a^, ^b^	4.8 (3.9–6.0)^a^, ^b^, ^c^	<0.001
∼11 kDa	12.2 (7.2–23.8)	12.1 (9.5–17.2)	4.4 (2.9–9.0)^a^, ^b^	1.2 (0.6–2.1)^a^, ^b^, ^c^	<0.001
∼65 kDa	5.5 (3.3–10.3)	7.2 (5.3–11.1)	10.8 (6.5–18.5)^a^	14.8 (9.2–19.3)^a^, ^b^	<0.001
∼85 kDa	1.7 (1.2–2.9)	3.1 (2.5–5.8)	11.7 (8.2–17.5)^a^, ^b^	24.4 (16.2–35.5)^a^, ^b^, ^c^	<0.001
∼120 kDa	72.3 (62.6–78.3)	70.2 (64.8–76.2)	62.8 (56.8–76.5)	51.5 (41.1–65.8)^a^, ^b^	<0.001

Medians (interquartiles) are presented.

PCA, perchloric acid; TP, total protein; CD, Crohn's disease; Kruskal‐Wallis test with post hoc analysis was used to compare groups. Supersript lowercase letters indicate the significance of post hoc analysis.

a *p* < 0.01 when compared to controls.

b *p* < 0.01 when compared to nonactive CD.

c *p* < 0.01 when compared to active CD patients.

### 2.3 SDS electrophoresis

Electrophoretic separation of PCA‐soluble proteins was carried out by 1‐dimensional sodium dodecyl sulfate polyacrylamide gel electrophoresis (1D SDS‐PAGE) according to the method of Laemmli 20. The stacking and resolving gels contained 5 and 7.5% (w/v) acrylamide/methylene‐bis‐acrylamide (30:0.8) solution, respectively. Briefly, 10 μL sample volumes were loaded and run at 190 V for 45 min in Mini‐PROTEAN® 3 system (Bio‐Rad Laboratories, Hercules, CA, USA). After electrophoresis, the protein patterns were detected on gel by silver staining using the method of Willoughby and Lambert 21.

### 2.4 Microchip gel electrophoresis

Electrophoresis in microchips was performed with the High Sensitivity Protein 250 LabChip kit in the commercially available Agilent 2100 Bioanalyzer system (Agilent Technologies, Waldbronn, Germany) equipped with a diode laser for fluorescence detection with 630 nm excitation and 680 emission wavelengths, as described previously 22. The kit included microchips and reagents, such as High Sensitivity Protein 250 Labeling Dye, DMSO, ethanolamine, Protein 250 Standard Labeling Buffer (300 mM Tris/HCl, pH >8.5), Gel Matrix (4.5% polydimethyl acrylamide‐based linear polymer solution at pH 8), Destaining Solution, and Sample Buffer. The denaturing solution containing SDS and dithiothreitol (DTT) was prepared by adding 3.5 μL 1 M DTT (Boehringer Mannheim GmbH, Mannheim, Germany) to 100 μL Sample Buffer. The protocol was optimized for PCA‐soluble proteins to achieve good separation and sensitivity. Briefly, fluorescently labeled proteins were prepared by mixing 5 μL sample volume with 0.5 μL diluted fluorescent dye/DMSO solution (the dye was 10 times diluted with water compared to the original protocol) and incubated for 10 min at room temperature (instead of the 30 min incubation on ice in the original protocol). The excess dye (i.e., the unbound dye) was quenched by adding 0.5 μL ethanolamine and incubated for 10 min at room temperature. The labeled samples were diluted five times by adding 24 μL deionized water (instead of a 200 times dilution in the protocol). Briefly, 4 μL of this diluted sample solutions were combined with 2 μL denaturing solution, incubated at 100°C for 5 min, and centrifuged. Briefly, 6 μL of each sample were loaded on the microchip channels filled with a polydimethyl‐acrylamide‐based linear polymer solution (pH 8). The respective well was loaded with the Destaining Solution.

Samples were injected with 1000 V for 80 s (injection volume was ca. 40 pL), and the separation was continued toward the anode at 1000 V for 60 s at 30°C. Each sample was analyzed at least three times. The molecular masses of the protein components were determined by using the calibration curves in 22. From the area under the curve (AUC) of the components, relative proportions were calculated and expressed as a percentage of the total AUC.

### 2.5 Statistical analyses

Statistical analysis was performed using the IBM SPSS statistical software version 22 (IBM, New York, NY, USA). The Kruskal–Wallis test with post hoc analyses was used for the comparison of the groups. The *p*‐values less than 0.05 were considered to be significant. All data were expressed as median (interquartiles).

## 3 Results and discussion

Examining the demographic, clinical, and laboratory data of the study population, we found that Crohn's patients and the healthy control group were similar in age and gender distribution (Table 1). In contrast, the mean age of the septic patients was higher compared with the other groups. The main reason for this is that sepsis is more common in the elderly subjects, who suffer from comorbidities, have poorer immune status, and require surgical interventions more frequently 23. The total protein content of the PCA extracts of healthy individuals does not depend on age and serum protein concentration; also, there is no correlation with creatinine, uric acid, and albumin levels (unpublished observations). It cannot be excluded that there might be some minor individual differences in the protein patterns even among healthy people; however, in systemic inflammation, both the amount of PCA soluble proteins and the MGE profiles show marked changes when compared with those of the controls.

Laboratory analysis is necessary for monitoring systemic inflammation, and it is of utmost importance to help clinical decision‐making, and for early recognition of complications 24, 25. Conventional inflammatory parameters (WBC, hs‐CRP, and ORM) are tested widely in daily routine. As expected, we found significant differences in these parameters between the four groups, as shown in Table 1. However, these parameters are not always capable for the early recognition of inflammatory activation. WBC is not sensitive enough and hs‐CRP and ORM are nonspecific 25, 26, therefore, novel laboratory approaches can have an important place in managing systemic diseases.

The PCA precipitation method is able to isolate and enrich low abundant molecules and glycoproteins, which are not detected usually in serum by performing other methods 2, 4, 27, 28.

According to our knowledge, this is the first study that investigates the PCA‐soluble proteins by high‐sensitivity electrophoretic methods in systemic inflammatory disorders and compares them with those of healthy individuals. This method, however, has no opportunity to associate the PCA‐soluble proteins with diseases as it has been shown in 2, 4, 5. Previously, we have verified by western blot technique that traces of albumin may occur in the acid soluble protein samples; however, the amount of the immune reactive albumin was negligible when compared with that of the untreated serum samples (data not shown). The total protein analyses of PCA‐soluble serum fractions showed higher concentrations in inflammatory conditions (Table 2). The amount of the PCA‐soluble proteins was significantly (*p* < 0.001) increased in active CD patients and in septic patients compared with nonactive CD and control individuals; however, data of nonactive CD patients did not differ from the control group. The elevated PCA‐soluble protein concentration might be related to systemic inflammation, since several heavily glycosylated serum proteins (ORM, haptoglobin, and alpha‐1‐antitrypsin) can remain soluble in PCA, and the PCA‐soluble fraction is presumably rich in inflammatory markers 5, 7. Also, previous studies have reported elevated PCA‐soluble proteins in malignancies 7. These results correlate well with the observations of altering glycosylation of acute phase proteins during diseases, which influence both the physicochemical properties and the immunomodulatory function 29, 30, 31, 32, 33, 34, 35, 36. Specific changes in glycosylation have been used as novel potential markers of inflammation and cancer 15, 37, 38, 39, 40. Moreover, previous studies demonstrated that various glycoforms of acute phase proteins correlate with the severity of the disease in certain pathophysiological conditions 41, 42, 43, 44.

**Figure 1:**
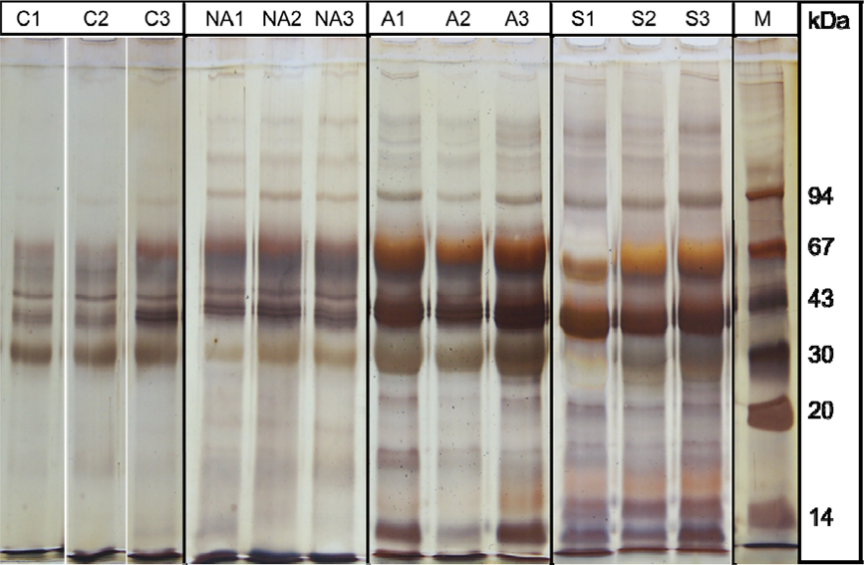
SDS‐PAGE gel images with silver staining of PCA‐soluble proteins. (A) control (C1–3), (B) nonactive Crohn's disease (NA1–3), (C) active CD (A1–3), and (D) sepsis (S1‐3). The SDS‐PAGE was run at 190V for 45 min; 10 μL sample was loaded. M, molecular mass marker.

In our study, the qualitative analyses of the PCA‐soluble serum fractions showed marked differences in the protein composition (healthy and inflammatory conditions). We observed striking differences of SDS‐PAGE patterns between healthy controls and patients with active systemic inflammation (active CD and sepsis) as seen on Fig. 1. More fractions could be detected on SDS‐PAGE by silver staining in the cases of active CD and in sepsis than in the cases of healthy individuals, and the gel images of nonactive CD patients seemed to be very similar to the controls. Nevertheless, there were no marked differences between the individual patterns within the groups (control, nonactive CD, active CD, and sepsis).

The SDS‐PAGE profiles show that a variation in the relative amounts exists between the different protein components. However, the SDS‐PAGE with silver staining is a laborious and time‐consuming method; moreover, the silver‐stained gels are not suitable even for semiquantitative evaluation because of the nonlinear nature of silver accumulation on the surface of the proteins 20.

In contrast to SDS‐PAGE, MGE is a miniaturized, rapid method allowing quantitative evaluation and high reproducibility 19, 22. Thus, the fine differences in the distribution and intensity of the PCA‐soluble bands could be detected and evaluated better by MGE analysis.

**Figure 2:**
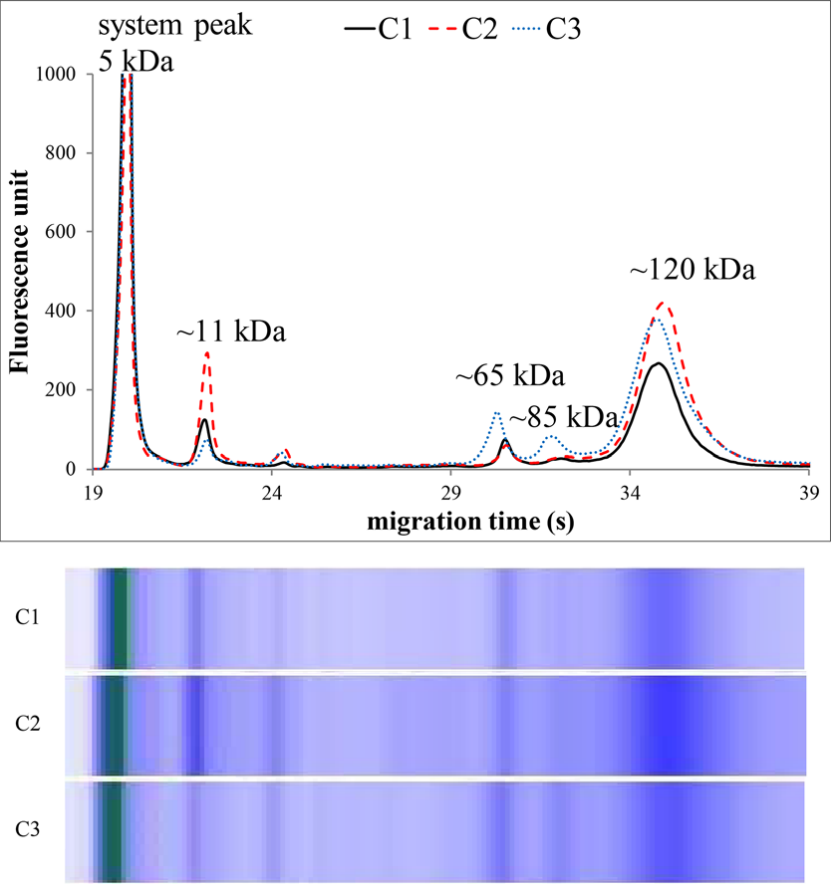
MGE electrophoretic profiles and gel‐like images of PCA‐soluble proteins in healthy controls (C1–3). Experimental conditions of the electrophoresis with the HSP 250 Protein Chip are described in the Materials and methods. The total protein concentration applied in the chip well was roughly 0.1 μg/μL.

**Figure 3:**
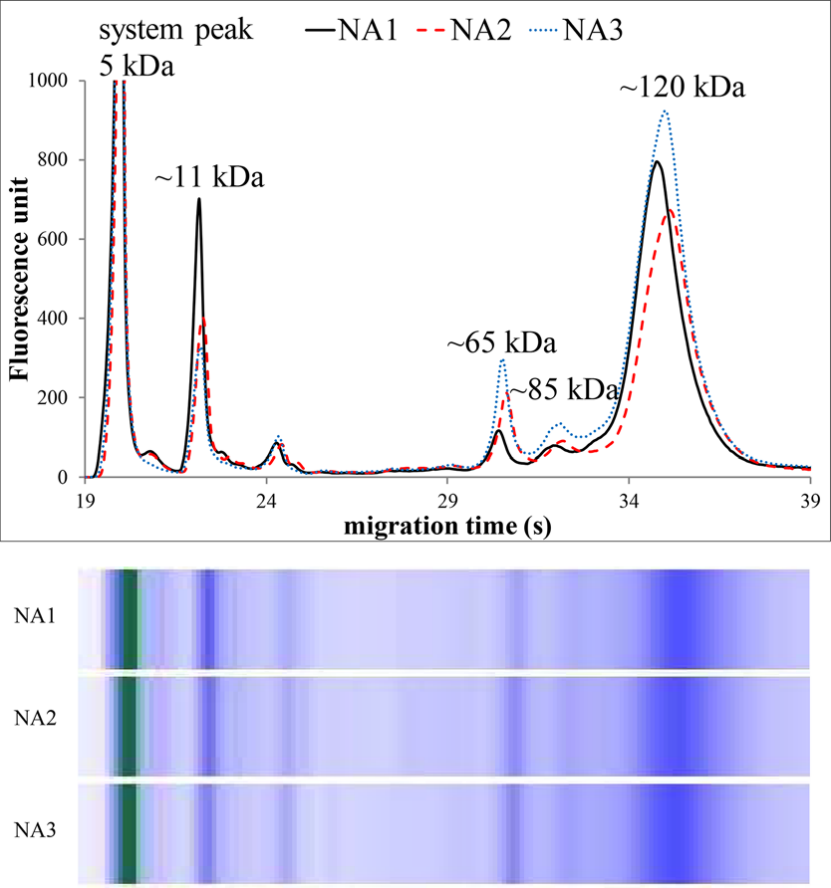
MGE electrophoretic profiles and gel‐like images of PCA‐soluble proteins in patients with nonactive Crohn's disease (NA1–3). The experimental conditions were the same as in Fig. 2. The total protein concentration applied in the chip well was roughly 0.2 μg/μL.

**Figure 4:**
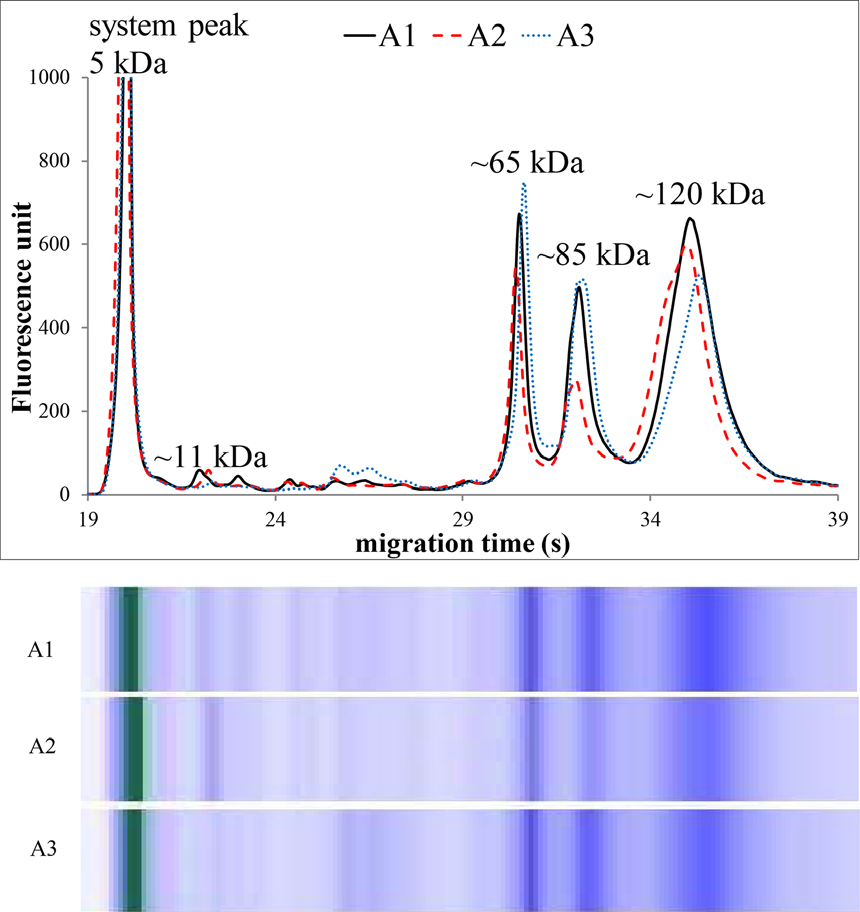
MGE electrophoretic profiles and gel‐like images of PCA‐soluble proteins in patients with active Crohn's disease (A1–3). The experimental conditions were the same as in Fig. 2. The total protein concentration applied in the chip well was roughly 0.3 μg/μL.

**Figure 5:**
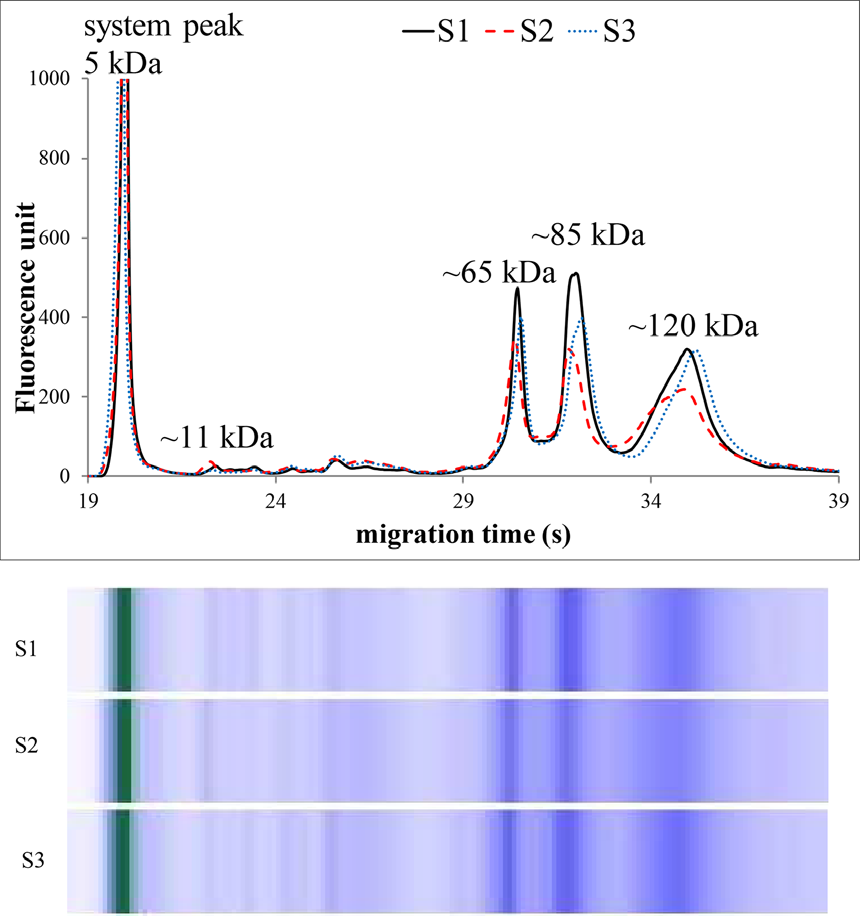
MGE electrophoretic profiles and gel‐like images of PCA‐soluble proteins in septic patients (S1–3). The experimental conditions were the same as in Fig. 2. The total protein concentration applied in the chip well was roughly 0.5 μg/μL.

Figures 2, 3, 4, 5 illustrate three representative samples for PCA‐soluble proteins from each patient‐group and the control group to show the characteristic patterns of the diseases.

The MGE of the labeled PCA‐soluble proteins showed characteristic profiles after the system peak (the fluorescent dye bound to ethanolamine) both for healthy controls and for patients suffering from inflammation.

Significant differences were found in the relative amounts of the protein components on MGE patterns, and we observed four characteristic bands with varying intensity at the ∼11, ∼65, ∼85, and ∼120 kDa regions (Figs. 2, 3, 4, 5, Table 2).

The relative amount of ∼11 kDa band (given as the percentage of the total protein content) was about three times higher in patients without inflammation (control and nonactive CD patients) compared with active CD patients. Moreover, this band was much less detectable in severe sepsis (*p* < 0.001). On the other hand, the proportions of the ∼65, ∼85, and ∼120 kDa bands were significantly higher in patients with acute active inflammation (active CD and septic patients) compared to nonactive and healthy patients (*p* < 0.001), as shown in Table 2 and Figs. 2, 3, 4, 5. The MGE profiles of patients with nonactive CD seem to be very similar to those of control individuals (Figs. 2 and 3), while the patterns of active CD patients show similarity to those of septic patients (Figs. 4 and 5).

The profiles in the MGE, similarly to SDS‐PAGE, show the quantitative and qualitative differences in the PCA‐soluble protein composition in systemic inflammatory conditions; however, to date, there is only limited evidence for the exact constituents and characterization of the proteins. Probably, different heavily glycosylated serum acute phase proteins, such as ORM, alpha‐1‐antitrypsin, complement factors, etc., might be responsible for the observed electrophoretic patterns in inflammation, as it has been demonstrated in cancer patients recently 5. Furthermore, the changes in the carbohydrate structures of glycoproteins during inflammation might also cause diverse electrophoretic profiles 29, 31, 33.

## 4 Concluding remarks

To conclude, the marked differences in acid‐soluble proteins observed in patients with ongoing systemic inflammation might be a good indicator of inflammation and can be of high importance in medical laboratory practice. The amounts of acid‐soluble proteins increased, and characteristic protein patterns were observed, which seem to be capable of monitoring systemic inflammatory activation. However, the exact protein composition of PCA‐soluble serum fraction has not been explored yet, and further proteomic studies should investigate and identify PCA‐soluble protein markers, which can be useful in daily patient care.

*The authors have declared no conflict of interest*.
